# Supplementary material for: Massive proliferation of retrotransposons contributes to genome size expansion in species of the Pseudocercospora genus
Source: Mob DNA. 2026 Feb 26;17:11. doi: 10.1186/s13100-026-00396-x (PMC13041039; doi:10.1186/s13100-026-00396-x)
Supplement: Supplementary file 1 — Supplementary Material 1. [file 13100_2026_396_MOESM1_ESM.pdf]

SUPPLEMENTARY INFORMATION

Massive proliferation of retrotransposons contributes to genome size expansion in species of the *Pseudocercospora* genus

González Sáyer Sandra-Milena et al.

Supplementary Tables

(see separate file)

Supplementary Table S1: Accession numbers and data resources for the genomes analyzed in this study.

Supplementary Table S2: *Pseudocercospora ulei* isolates analyzed in this study.

Supplementary Figures

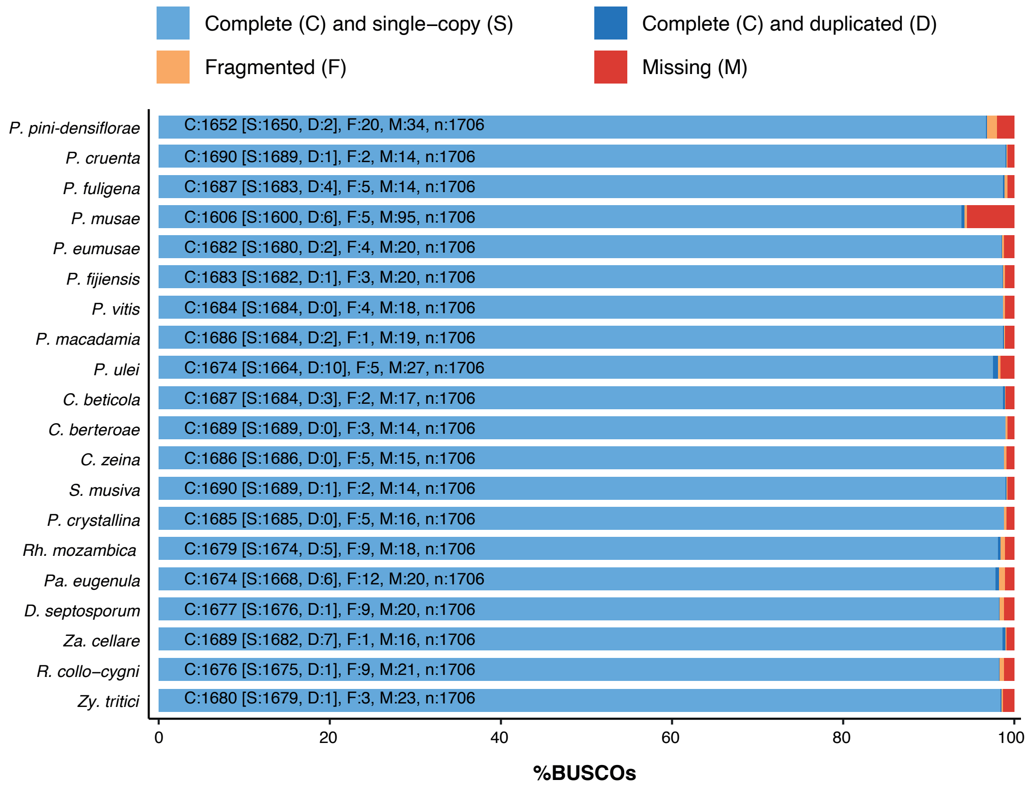

Supplementary Figure S1. Detailed BUSCO (Benchmarking Universal Single-Copy Orthologs) scores for Mycosphaerellaceae genome assemblies. 1706 BUSCO orthologs from the ascomycota_odb10 database were analyzed, and the complete (single copy or duplicated), fragmented and missing orthologs were listed.

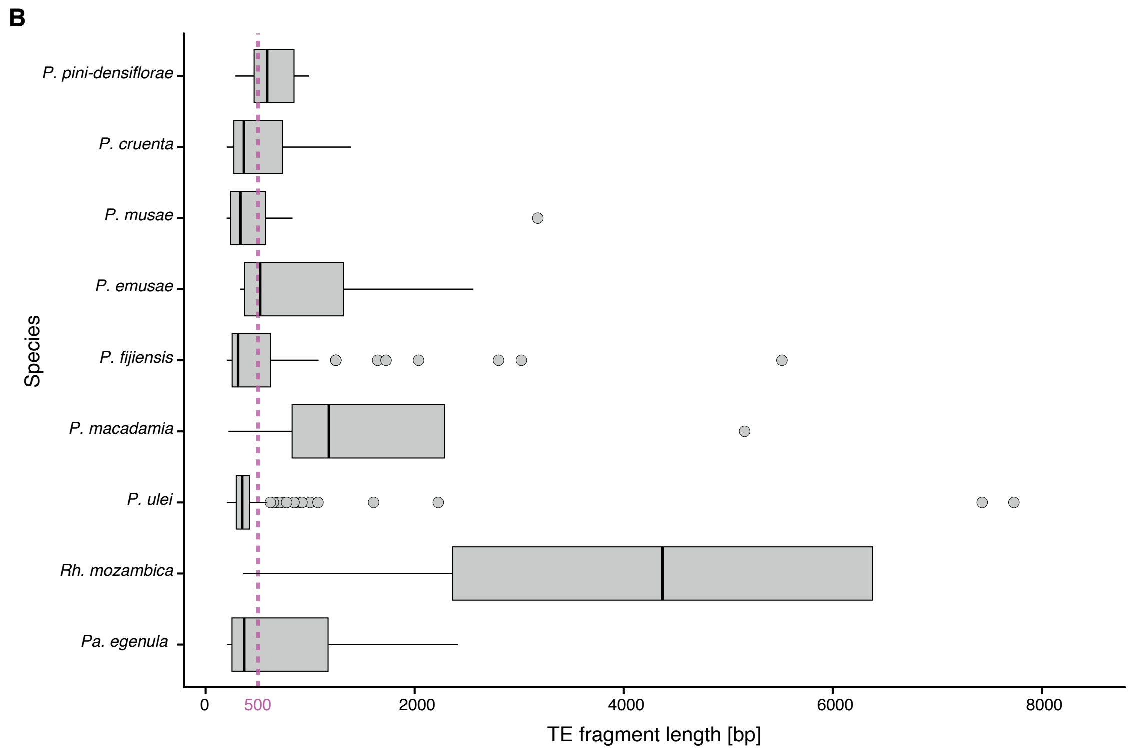

Supplementary Figure S2. Repeat element lengths in the seven strains with the most expanded genome size of the *Pseudocercospora* genus and two closely related species. Length distribution of TE fragments per genome. The mauve line indicates 500 bp.

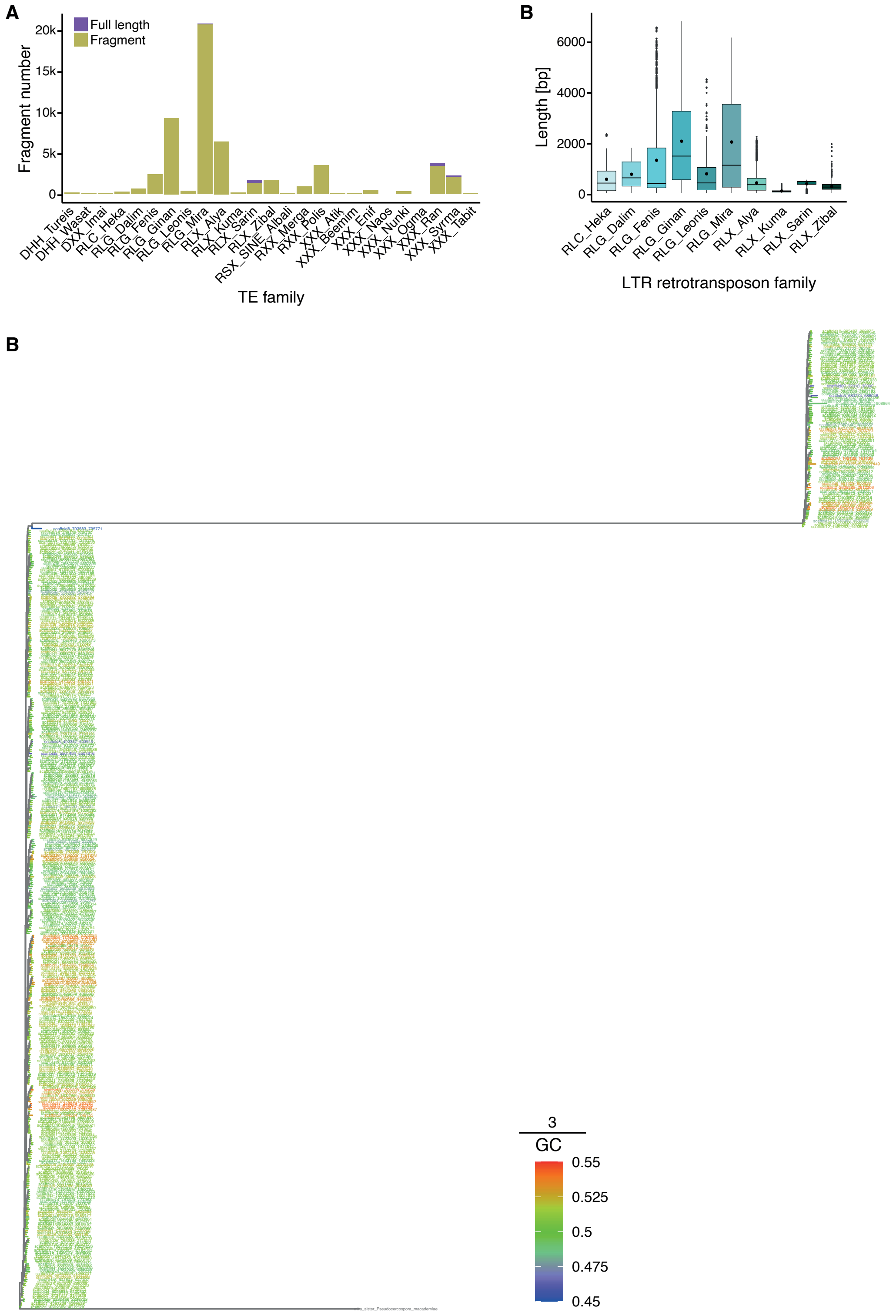

Supplementary Figure S3. Manually curated TE families in *P. ulei*. A) Copy numbers of TE families. The color indicates if the length of the TE copy was the same (or shorter by >20%) as the consensus. B) Length distribution of TEs for retrotransposons. C) Phylogenetic tree for the coding regions of the RLG_Mira family. The color indicates the GC content, with a low GC content (blue) indicating a potential impact of RIP.

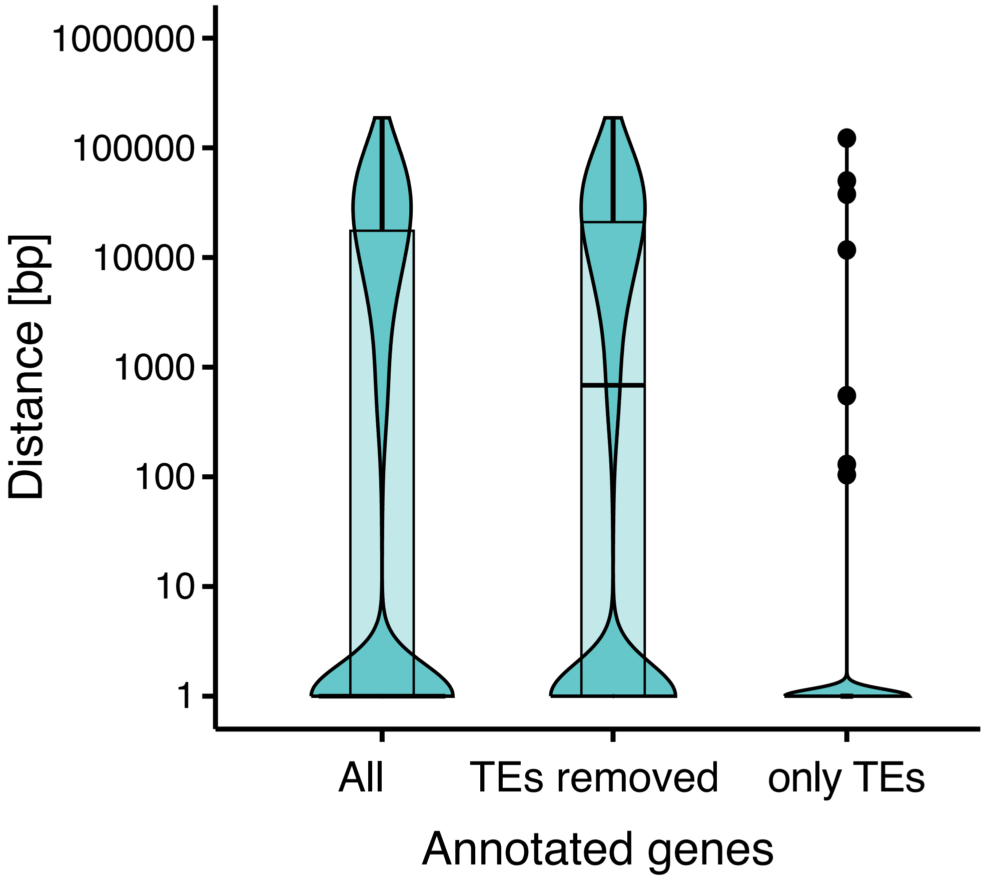

Supplementary Figure S4. Variation in distances between genes and the closest retrotransposon. The distribution was analyzed across three datasets: 1) all annotated genes, 2) a subset of genes identified without any evidence of TE overlap and 3) genes with evidence for TE overlaps ("only TEs"). Genes with more than one TE insertion were treated as a single insertion.
